# Fungal Communities Associated with Wooden Coffins in a Prehistoric Burial Cave

**DOI:** 10.3390/jof12050380

**Published:** 2026-05-21

**Authors:** Nantana Mills, Natasha Mills, Nakarin Suwannarach, Nuttapol Noirungsee, Jaturong Kumla, Sahutchai Inwongwan, Rujipas Yongsawas, Chanon Saksunwiriya, Varis Domethong, Rasmi Shoocongdej, Terd Disayathanoowat

**Affiliations:** 1 Department of Biology, Faculty of Science, Chiang Mai University, Chiang Mai 50200, Thailand; nantana.mills8@gmail.com (N.M.); natasha.mills102@gmail.com (N.M.); nakarin.su@cmu.ac.th (N.S.); nuttapol.n@cmu.ac.th (N.N.); jaturong_yai@hotmail.com (J.K.); sahutchai.inwongwan@cmu.ac.th (S.I.); r.yongsawas@gmail.com (R.Y.); chanon_viriya@hotmail.com (C.S.); 2 Center of Excellence in Microbial Diversity and Sustainable Utilization, Faculty of Science, Chiang Mai University, Chiang Mai 50200, Thailand; 3 Department of History, Faculty of Humanities, Chiang Mai University, Chiang Mai 50200, Thailand; varis.dome@gmail.com; 4 The Conservation of Ancient Log Coffins of Phi Man Long Long Rak Cave, Mae Hong Son Province Project, Princess Maha Chakri Sirindhorn Anthropological Centre, 20, Boromrachachonnani Road, Taling Chan, Bangkok 10170, Thailand; rasmis@gmail.com; 5 Department of Archeology, Faculty of Archeology, Silpakorn University, Bangkok 102000, Thailand

**Keywords:** biodeterioration, cave microbiology, fungal communities, metagenomic

## Abstract

Phi Man Long Long Rak Cave, located in Mae Hong Son Province, northern Thailand, is a prehistoric burial site containing ancient wooden coffins that have undergone biodeterioration, likely due to fungal activity. Both culture-dependent and culture-independent approaches were employed to characterize fungal communities and assess their roles in wood degradation. Culture-dependent analysis identified five *Aspergillus* isolates from the wooden coffins, most of which produced cellulolytic and hemicellulolytic enzymes; some isolates also produced organic acids, indicating significant degradative potential. Culture-independent analysis revealed a community dominated by *Aspergillus,* together with additional taxa such as *Penicillium* and *Ceriporia* that were not detected by cultivation, highlighting greater community diversity and demonstrating the complementarity of the two methods. Functional prediction indicated a predominance of saprotrophic fungi. The presence of shared dominant taxa between soil and coffin-associated substrates suggests ecological connectivity at the soil–coffin interface, although the direction of dispersal cannot be determined from the present data. All tested fungicides inhibited fungal growth, with the highest efficacy observed in the formulation containing the highest proportion of active components. Taken together, these findings provide insights into fungal biodeterioration processes and inform conservation strategies.

## 1. Introduction

Caves are important archaeological sites that provide a critical foundation for understanding the history and development of human civilization. Across the world, caves have frequently served as archaeological burial sites, preserving human remains and associated cultural materials. In Thailand, one particularly remarkable example is Phi Man Long Long Rak Cave, a prehistoric site estimated to be around 2120 years old, located in Mae Hong Son Province, northern Thailand [1,2]. This cave functions as a burial site for prehistoric communities who inhabited the area and is especially notable for its association with the so-called “wooden coffin culture”. This culture is characterized by the use of intricately crafted wooden coffins rather than direct in-ground burials [1,2].

Within the cave, numerous complete and fragmentary wooden coffins, together with wooden debris believed to have once formed part of coffins, as well as various associated artifacts have been discovered [1,2]. The coffins are coated and decorated with patterns made from natural wood resin, locally known as “Yang Rak” [1,2]. These findings highlight the site’s cultural and archaeological significance and make it an important location for studying prehistoric burial practices, lifestyles, and interactions among ancient people, particularly those related to wooden culture and wooden heritage. However, many wooden coffins have deteriorated over time, and without adequate conservation measures, this valuable wooden cultural heritage is at risk of further degradation.

Deterioration of wooden coffins in cave environments is driven by a combination of physical, chemical, and biological factors. These include abiotic factors, such as environmental conditions, as well as biotic factors, particularly microorganisms such as fungi and bacteria [3,4,5]. Although both fungi and bacteria contribute to wood degradation, their relative significance depends on environmental conditions and the specific properties of the wood. Bacteria are typically associated with the degradation of waterlogged or submerged wood, where elevated moisture levels and low oxygen promote bacterial activity [3,5]. In contrast, fungi are more frequently associated with the degradation of wood in terrestrial, above-ground environments, and in wood in contact with soil, where oxygen is more readily accessible [3,4,5].

In terrestrial cave environments, wooden coffins and other preserved wooden heritage objects are typically exposed to air and remain in close contact with the ground rather than being waterlogged. Under these conditions, fungi are expected to play a leading role in the wood deterioration process, whereas bacteria may still contribute but are likely to be of lesser importance. Fungi cause biodeterioration through hyphal penetration, wood-degrading enzymes, and the production of organic acids that weaken the wood structure [3,4,6,7,8]. Genera such as *Aspergillus* and *Penicillium*, which are commonly reported in cave and wood substrate environments [6,9,10,11,12,13,14,15], are known as wood-decay fungi species with the ability to attack wood cell walls through the erosion and degradation of wood structure components [3,6,10], suggesting that fungi present in cave environments are also involved in wood biodeterioration. In addition, environmental conditions such as temperature, relative humidity, nutrient availability, and substrate properties further act as filters that influence fungal colonization and biodeterioration rates by shaping fungal growth, activity, and water-associated decay processes [3,8,16,17,18].

Tropical cave environments are characterized by persistently high humidity and stable temperature ranges, conditions that strongly favor fungal establishment and activity [9,19,20]. Consequently, tropical caves often experience fungal growth and colonization [19]. Similarly, at Phi Man Long Long Rak Cave, the Silpakorn University conservation team observed visible fungal growth on wooden coffins, in coffin-associated soil, and on coffin-associated substrates such as artificial support surfaces, suggesting active fungal colonization [1,2]. However, it remains unclear whether coffin-associated fungi originate primarily from specific environmental substrates within the cave, such as soil, cave walls, or other surrounding substrates. Identifying these potential environmental reservoirs is important for understanding how fungal colonization occurs on wooden artifacts preserved in caves.

Although fungi associated with wooden cultural heritage have received increasing attention in recent years [4,6,21,22,23], many studies have relied primarily on culture-dependent approaches that isolate fungi directly from wooden materials [6,22,23]. While these methods provide valuable fungal isolates and allow for direct testing of their wood-degrading abilities, they do not reveal the full fungal community profile present in the environment. The integration of fungal community structure and the ecological mechanisms underlying wood biodeterioration in cave-preserved wooden heritage remains limited. The functions of cave fungal communities at each stage of wood biodeterioration, especially at the early stage that initiates deterioration, are poorly understood. The origins of fungi linked with wood decay in caves, whether they come from the wood itself or from environmental reservoirs such as soil or associated substrates, also remain unclear.

To address this gap, this study employs both culture-dependent and culture-independent approaches to advance understanding of fungal community structure in wooden coffins and the cave environment. This study aims to investigate the ecological mechanisms shaping fungal communities, with particular emphasis on those colonizing wooden coffins and contributing to early-stage biodeterioration. The objectives are to identify dominant fungi associated with wooden coffins, characterize fungal communities in the cave environment, assess their biodeterioration potential, and determine the environmental reservoirs that may serve as sources of coffin-associated fungi. This integrated framework provides a comprehensive understanding of fungal-driven biodeterioration and its stages, including the potential origins of fungal colonization. Furthermore, the findings inform the foundation and development of improved conservation and preservation strategies for cave-preserved wooden heritage and contribute to broader knowledge of the ecology of wood-associated fungi.

## 2. Materials and Methods

### 2.1. Study Site and Sampling

#### 2.1.1. Study Site

**Figure 1:**
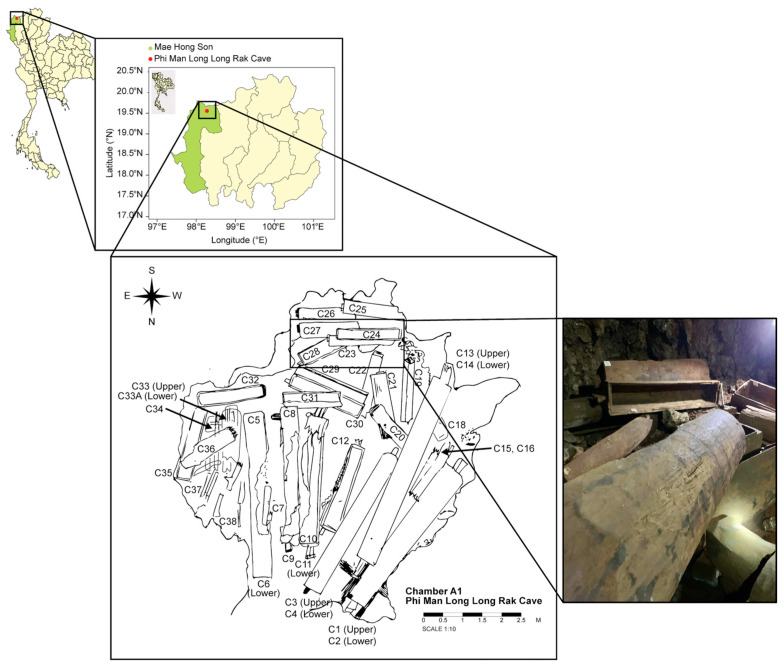
Map of the sampling site, Phi Man Long Long Rak Cave, Mae Hong Son Province, northern Thailand. The figure shows the location of the cave on the map of Thailand, a sketch of Chamber A1, and a photograph of a wooden coffin found at the site.

The sample site, Phi Man Long Long Rak Cave, is located in the Tham Lot subdistrict, Pang Mapha district, Mae Hong Son Province, northern Thailand (19°33′21.1″ N, 98°16′28.0″ E). The cave is a 2120-year-old prehistoric burial site situated within a limestone mountain, surrounded by bamboo and a mixed deciduous forest (Figure 1) [1,2].

**Figure 2:**
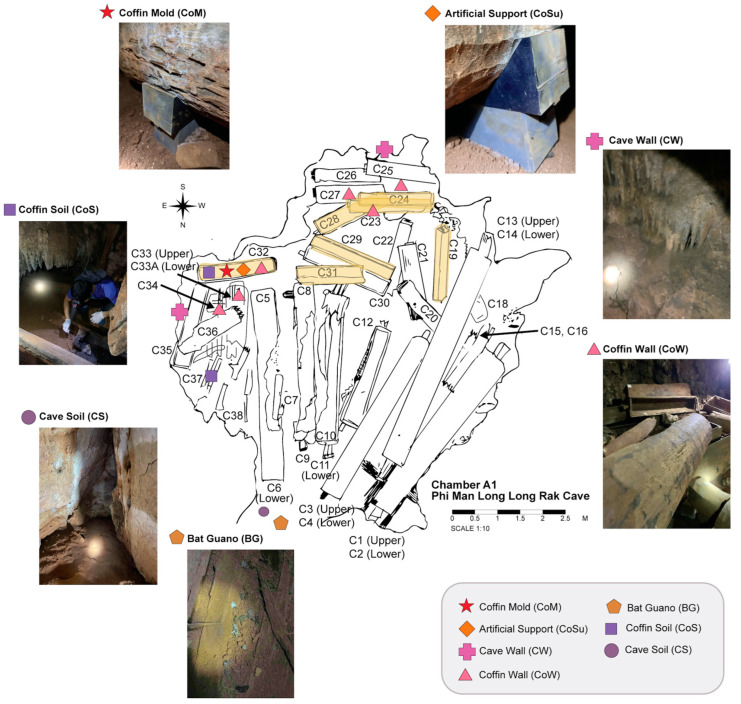
Map of sampling points. Chamber A1 with the locations of sampling points in 2023 and 2024. Each colored shape represents a different sample group; the coffin highlighted in yellow indicates fungal colonization. CS: cave soil, CoS: coffin soil, CW: cave wall, CoW: coffin wall, CoM: coffin mold, CoSu: artificial support, and BG: bat guano.

The cave comprises three main chambers: A (A1, A2), B, and C. Chamber A1, which contains the highest number of wooden coffins among all the chambers, was selected as the focal site of this study. The chamber is a wide, dark area located in the innermost part of the cave. Its entrance is on the north side, and it extends in a north–south direction, measuring approximately 8 m wide and 9 m long. Moreover, extensive fungal colonization has been observed throughout this chamber, particularly on coffin surfaces and on surrounding substrates, such as artificial supports (Figure 2) [1,2]. These characteristics make Chamber A1 suitable for investigating fungal community structuring and wood biodeterioration processes.

#### 2.1.2. Sample Collection

The sample collection was divided into two parts: one for culture-dependent isolation and biodeterioration assessment of wooden coffin fungi, and the other for culture-independent analysis of environmental substrates as potential sources of coffin-associated fungi. Samples were specifically selected from coffins with open lids to allow sampling of both interior and exterior surfaces. For fungal isolation and biodeterioration assessment, wooden coffin fragments were collected from various parts of coffin number C32, including the lid, body, and coffin tag paper. These samples served as independent biological sources associated with the coffin and its constituent material. All samples were stored in sterile containers.

For environmental substrate sampling, seven environmental substrate groups within Chamber A1 were collected. These comprised cave soil (CS, *n* = 3), coffin soil (CoS, *n* = 5), the cave wall surface (CW, *n* = 5), the coffin wall surface (CoW, *n* = 6), the coffin wall surface with visible mold (CoM, *n* = 4), the artificial support surface (CoSu, *n* = 3), and bat guano (BG, *n* = 2) (Figure 2). Collectively, these substrates represent five environmental groups within the chamber, including soil (CS and CoS), cave wall (CW), coffin (CoW and CoM), artificial support (CoSu), and bat guano (BG). The soil and bat guano samples were scooped from the soil within the chamber and around the coffin areas, and stored in sterilized 1.5 mL microcentrifuge tubes. In contrast, the surface samples were collected by gently swabbing the surfaces with sterile cotton swabs and stored in sterilized 50 mL microcentrifuge tubes. All environmental substrate samples were stored in DNA/RNA Shield reagent (Zymo Research, Freiburg im Breisgau, Germany) and then stored at −20 °C until DNA extraction for next-generation sequencing (NGS).

The sample collection period was divided into two phases. The first phase took place on 1 April 2023, during which wooden coffin fragments and five substrate samples were collected, including CS, CoS, CW, CoW, and BG (Figure 2). The second phase took place on 25 April 2024, and two additional substrate samples, CoM and CoSu, were collected (Figure 2). Detailed information on seven environmental substrates and wooden coffin samples in Chamber A1 is provided in Supplementary Table S1.

#### 2.1.3. Environmental Parameters

Environmental parameters, including temperature and relative humidity, were recorded in Chamber A1 during the sample collection periods on 1 April 2023 and 25 April 2024. Temperature and relative humidity were measured at a single point using a HOBO Temperature/Relative Humidity Data Logger (HOBO, Bourne, MA, USA), which was installed in the chamber and programmed to record data at 30 min intervals. The environmental values reported in this study represent averages of continuous measurement data obtained during the sample collection periods.

### 2.2. Culture-Dependent Isolation and Biodeterioration Assessment of Wooden Coffin Fungi

Wooden coffin fragments collected from Chamber A1 were processed using culture-dependent methods to isolate viable fungi colonizing the wooden coffins and to evaluate their biodeterioration potential.

#### 2.2.1. Fungal Isolation and Identification

Wooden coffin fragment samples were isolated using a single-spore isolation technique [24], with all isolation processes performed under a stereomicroscope. After the spores germinated, each germinated spore was transferred to a potato dextrose agar (PDA) plate, with five germinated spores placed on each plate. The plates were then incubated at room temperature for 3–5 days, or until the colony diameter reached approximately 1–2 cm. The different characteristics of the fungal colonies were observed and recorded. Finally, each fungal colony was picked and transferred to a new PDA plate to obtain a pure single colony. These pure colonies were then used for further testing for biodeterioration properties. For molecular identification, genomic DNA was extracted from each pure isolate according to the manufacturer’s instructions for the DNA Extraction Mini Kit (FAVORGEN, Pingtung, China). The internal transcribed spacer (ITS), β-tubulin (*BenA*), and calmodulin (*CaM*) were amplified using the primer pairs ITS5/ITS4, Bt2a/Bt2b, and CMD5/CMD6, respectively. PCR amplifications were performed with an initial denaturation at 94 °C for 5 min, followed by 35 cycles of denaturation at 94 °C for 45 s; annealing at 52 °C for 45 s for ITS or 55 °C for 45 s for *BenA* and *CaM*; and extension at 72 °C for 1 min, with a final extension at 72 °C for 10 min. PCR products were sent to the 1st BASE company (Kembangan, Malaysia) for sequencing. The resulting sequences were compared to those in the NCBI GenBank database using BLAST (https://blast.ncbi.nlm.nih.gov/Blast.cgi, accessed on 6 February 2026) to determine the closest taxonomic classifications. Taxonomic identification was based on the highest sequence similarity.

#### 2.2.2. Screening of Biodeterioration Properties

To evaluate mechanisms contributing to structural wood degradation, a fungal isolate was screened for enzymatic and acid-production activities involved in the degradation of cellulose, hemicellulose, lignin, and extractives (e.g., fats and waxes), the structural components of wood [6,25].

##### Wood-Degrading Enzyme Assays

The production of wood-degrading enzymes was screened for cellulase, mannanase, laccase, and lipase activities, representing the cellulolytic, hemicellulolytic, ligninolytic, and lipolytic enzyme groups, respectively [6,8,25,26,27]. Enzyme production by each fungal isolate was evaluated using a basal medium modified from Naresh et al., 2019 [28], supplemented with specific substrates for each assay: carboxymethyl cellulose (CMC) for cellulase, locust bean gum for mannanase, guaiacol for laccase, and Tween 20 for lipase (Table S2). The pH of each medium was adjusted to 7.0 with 1 M NaOH. An agar plug measuring 5 mm in diameter from the fungal isolate was placed at the center of the medium plate, with four replicates per isolate, and incubated at 25 °C for 12 days. Enzyme activity was assessed by visual observation of substrate degradation. Clear hydrolysis zones after staining with 1% Congo red, followed by destaining with 1 M NaCl, indicated cellulase and mannanase activity; reddish-brown zones indicated laccase activity; and clear or precipitate zones indicated lipase activity.

##### Acid Production Assay

The acid-producing capacity of the fungal isolate was evaluated by monitoring pH changes in potato dextrose broth (PDB). The initial pH of the broth was adjusted to 7.0 using 1 M NaOH. The fungal isolate was inoculated into individual culture tubes, with three replicates per isolate. The tubes were incubated at 25 ± 2 °C in a shaking incubator at 110 rpm for 5 days. After incubation, the broth was filtered, and the pH of the resulting filtrate was measured using a calibrated pH meter. The pH values were reported as mean ± standard deviation (SD).

### 2.3. Culture-Independent Analysis of Fungi in Various Locations Within the Cave

Seven environmental substrate samples collected from Chamber A1, described in Section 2.1.2, were analyzed using culture-independent methods to investigate fungal community composition, assess functional roles associated with wood deterioration mechanisms, and identify potential environmental reservoirs that may act as sources of fungi structuring coffin-associated fungal communities.

#### 2.3.1. Genomic DNA Extraction and Amplification for Sequencing

The ZymoBIOMICS™ DNA Miniprep Kit (Zymo Research, Freiburg im Breisgau, Germany) was used to extract DNA from seven environmental sample groups of samples collected from Chamber A1, following the manufacturer’s instructions. For each sample group, the amount of sample used varied depending on its physical form. A total of 0.25 g of the sample was used for soil and bat guano, while 1 mL of sample preserved in DNA/RNA Shield™ was used for cave wall surfaces, wooden coffin surfaces, wooden coffin surfaces with visible mold, and artificial support surfaces. Then, the purity, quality, and quantity of the extracted DNA were confirmed by measuring the DNA with a NanoDrop UV–Vis spectrophotometer (Thermo Fisher Scientific, Madison, WI, USA) and performing polymerase chain reaction (PCR) using a T100™ Thermal Cycler (Bio-Rad, Bangkok, Thailand). After confirmation, all extracted DNA samples were sent to Macrogen, Inc. (Geumcheon-gu, Seoul, Republic of Korea) for Illumina MiSeq sequencing.

#### 2.3.2. ITS Amplicon Sequencing for Metagenomic Analysis

Quantitative Insights Into Microbial Ecology 2 (QIIME2), version 2023.9, was used to analyze the fungal communities in each sample group. In QIIME2, raw NGS reads (FASTQ files) of all fungal DNA sequences were processed. Primer and adapter removal was performed using the ITS region with the forward primer ITS1 (5′-TCCTAGGTGAACCTGCGG-3′) and the reverse primer ITS2 (5′GCTGCGTTCTTCATCGATGC-3′) [29]. Then, denoising was performed using the DADA2 plugin, which included quality filtering, the removal of low-quality reads and chimeric sequences, and the merging of forward and reverse sequences. Subsequently, singleton removal was performed to exclude sequences that appeared only once, minimizing potential sequencing errors. Following this, rarefaction analysis was performed by generating rarefaction curves and normalizing sequencing depth across samples to assess the formation of plateau curves, ensuring adequate sequence reads. Finally, taxonomic classification was performed using the UNITE database, version 8.99, with a 99% identification confidence level. Fungal community compositions were visualized using pie charts showing the genera detected in each sample group. Genera with a relative abundance greater than 1% were shown individually, while those with less than 1%, along with uncultured and unidentified genera, were excluded. The low-abundance genera constituted a negligible portion of the dataset, with many occurring at less than 0.1%, and were omitted to improve clarity and emphasize dominant community members. Differences in fungal community composition among environmental substrates were evaluated using a one-way PERMANOVA test [30,31] based on the Bray–Curtis similarity index [32], with 9999 permutations and a significant differences threshold of *p* < 0.05, using the ‘Vegan’ package in RStudio, version 4.3.2.

#### 2.3.3. Venn Diagram Analysis

The fungal communities of fungal genera with a relative abundance greater than 1% from the seven environmental substrates within Chamber A1 were consolidated into five environmental groups, including soil, cave, coffin, artificial support, and bat guano, for Venn diagram analysis. The soil group included CS and CoS samples; the cave group included a CW sample; the coffin group included CoW and CoM samples; the artificial support group included a CoSu sample; and the bat guano group included a BG sample. This grouping approach was used to identify shared and unique genera across environmental substrates and to assess the overlap between coffin-associated communities and candidate environmental sources. The analysis was performed using the ‘VennDiagram’ package in RStudio, version 4.3.2.

#### 2.3.4. Functional Prediction Analysis

The fungal communities from the seven environmental substrates within Chamber A1 were consolidated into five environmental groups, including soil, cave, coffin, artificial support, and bat guano, the same as the groups of the Venn diagram analysis in Section 2.3.3 for functional prediction analysis. This approach was conducted to estimate ecological roles and functional potential related to wood deterioration. In particular, this approach was designed to identify which fungal communities within the environmental groups exhibited the greatest potential to contribute to wood-decay capability, thereby supporting inferences about potential environmental source contributions to wood-associated fungal colonization. This analysis was performed using two tools, including FungalTraits version 1.2.0 [33] and PICRUSt2 (Phylogenetic Investigation of Communities by Reconstruction of Unobserved States) version 2.4.1 [34]. FungalTraits was used to assess fungal ecological traits, functional characteristics, and lifestyle roles within the ecosystem; unidentified genera were excluded from this analysis. In parallel, PICRUSt2 was used to predict the production of wood-degrading enzymes that are involved in wood modification and degradation. The results from FungalTraits and PICRUSt2 analyses were visualized as a stacked bar plot and a heatmap, respectively, using the ‘ggplot2’ and ‘pheatmap’ packages in RStudio, version 4.3.2 [35,36].

#### 2.3.5. DNA Sequence Deposition

The raw ITS amplicon sequencing data from the seven environmental substrate groups are available in the National Center for Biotechnology Information (NCBI) under the BioProject accession number PRJNA1310159.

### 2.4. Commercial Fungicide Sensitivity and Conservation Implications

Commercial fungicide sensitivity assays were performed on a fungal isolate from wooden coffins to evaluate potential chemical treatments and inform appropriate conservation and preservation strategies.

#### Commercial Fungicide Sensitivity Test

Three commercial fungicides, namely Brand 1, Brand 2, and Brand 3, were selected to evaluate antifungal efficacy. All three commercial fungicides contained benzalkonium chloride as the active ingredient, while Brand 3 additionally contained a mixture of quaternary ammonium compounds and 2-octyl-3-isothiazolinone. Detailed information on the commercial fungicide component is provided in Supplementary Table S3. The antifungal efficacy of each commercial fungicide was evaluated using the paper disc diffusion method at four different concentrations, 100%, 50%, 25%, and 12.5%, modified from Li et al. [37] and Han et al. [8]. Fungal isolates were inoculated on a half-strength PDA medium using the swab technique. A sterile paper disc with a diameter of 8 mm was saturated with 50 µL of the respective anti-mold product and placed onto the medium plate in three replicates, and a control disc saturated with 50 µL of sterile deionized water was included for comparison. Then, the plates were incubated at 30 °C for 24 h; after that, the inhibition zones around the discs were measured and recorded. The significant differences (*p* < 0.05) between each commercial fungicide were assessed using a one-way ANOVA followed by Tukey’s HSD test.

## 3. Results

### 3.1. Environmental Parameters

During the sample collection periods (1 April 2023 and 25 April 2024), the temperature in Chamber A1 remained stable at 23 °C, with a relative humidity of approximately 70%. These conditions indicate consistent humidity, with low and stable temperatures during the sampling periods.

### 3.2. Culture-Dependent Isolation and Biodeterioration Properties of Wooden Coffin Fungi

#### 3.2.1. Fungal Isolation and Identification

Five pure fungal isolates, including A1, A1-2, A2, A2-2, and A2-3/1, were obtained from a wooden coffin fragment in chamber A1. Isolate A1 was from the coffin’s tag paper, isolates A2 and A2-3/1 were from the coffin body, and isolates A1-2 and A2-2 were from the coffin lid. Based on morphological investigation, all fungal isolates were initially identified as belonging to the genus *Aspergillus*. Therefore, their species identification was confirmed using ITS, *BenA*, and *CaM* sequence data. The sequences obtained from the fungal isolates in this study were deposited in the GenBank database (Table 1). Based on sequence similarity, isolates A1, A1-2, A2, A2-2, and A2-3/1 were identified as *A*. *terreus*, *A*. *fischeri*, *A*. *wentii*, *A*. *terreus*, and *A*. *sclerotiorum*, respectively.

#### 3.2.2. Screening of Biodeterioration Properties

**Figure 3:**
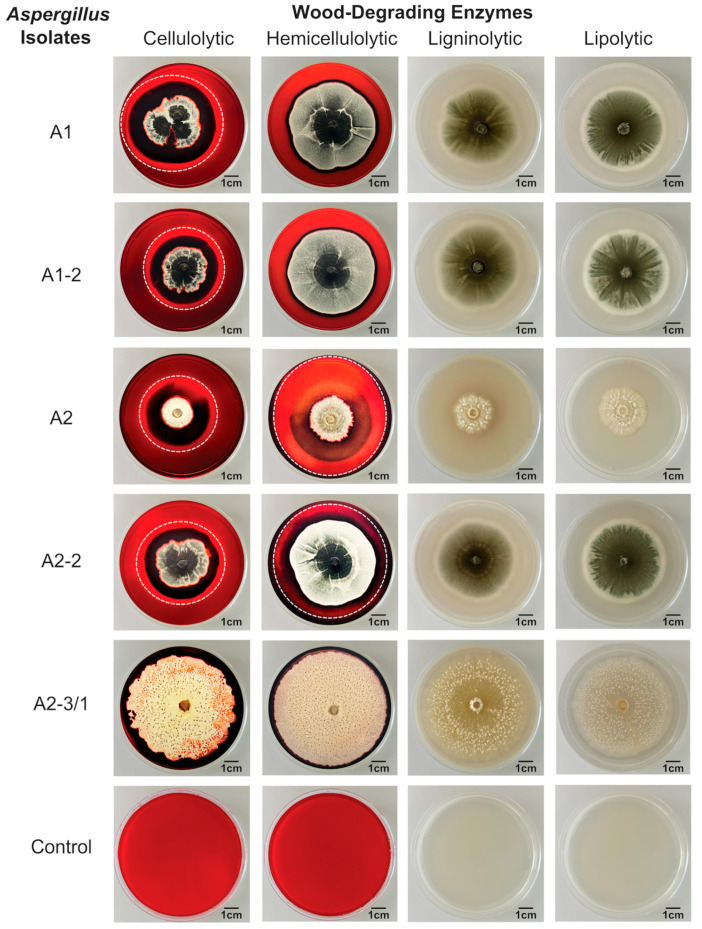
Effect of enzyme activity of wood-degrading enzymes on the growth of a fungal isolate on substrate-containing medium. The white dashed line in the cellulolytic (cellulase) and hemicellulolytic (mannanase) enzyme plates indicates the hydrolysis zone, while plates without a line represent complete hydrolysis across the plate. Ligninolytic (laccase) and lipolytic (lipase) enzymes showed no activity. A1: *Aspergillus terreus*, A1-2: *Aspergillus fischeri*, A2: *Aspergillus wentii*, A2-2: A*spergillus terreus*, and A2-3/1: *Aspergillus sclerotiorum*.

Screening for wood-degrading enzyme production demonstrated that, after 12 days of incubation, four fungal isolates, including A1, A1-2, A2, and A2-2, exhibited both cellulase and mannanase activities, as indicated by the clear hydrolysis zones on carboxymethyl cellulose (CMC) and locust bean gum-containing media, respectively. These results confirm the presence of cellulolytic and hemicellulolytic activities. In contrast, none of the isolates displayed laccase and lipase activities, which represent ligninolytic and lipolytic activities, respectively, on guaiacol and Tween 20-containing media (Table 2 and Figure 3). Moreover, screening for acid production revealed differential acid production among isolates after 5 days of incubation. The control medium without fungal inoculation had a pH of 5.81 ± 0.07 (Table S4). Among the five fungal isolates, only two isolates, A1 and A2-3/1, reduced the medium pH, indicating acid production (Table 3). Isolate A1 lowered the pH to 3.40 ± 0.17, while isolate A2-3/1 lowered the pH to 4.30 ± 0.32 (Table S4). Collectively, these findings indicate that the fungal isolates primarily target cellulose and hemicellulose components of the wood and may contribute to the biodeterioration of wooden coffins through both enzymatic degradation and acid production.

### 3.3. Culture-Independent Analysis of Fungi in Various Locations Within the Cave

#### 3.3.1. Sequence Read Curation

After primer and adapter trimming, a total of 2,616,073 fungal amplicon sequence variants (ASVs) were obtained from the demultiplexed sequence counts summary, with a minimum of 29,640 and a maximum of 159,417 ASVs. After denoising, quality filtering, and the removal of low-quality reads and chimeric sequences, the total was reduced to 1,955,305 ASVs, with a minimum of 17,915 and a maximum of 120,170 ASVs, and 9444 unique fungal ASVs. The subsequent removal of singleton ASVs slightly reduced the total to 1,955,301 ASVs, with a minimum of 17,915 and a maximum of 120,169 ASVs, and 9440 unique fungal ASVs. Taxonomic classification and rarefaction analysis, based on the plateauing of rarefaction curves, and normalized sequencing depth across samples, resulted in a final total of 501,620 ASVs, with a minimum and maximum of 17,915 ASVs and 7892 unique fungal ASVs used for metagenomic analysis in the culture-independent study. Detailed information on each processing step is provided in Supplementary Table S5 and Figure S1.

#### 3.3.2. Fungal Communities Within Chamber A1

In Chamber A1 of Phi Man Long Long Rak Cave, the seven environmental substrate groups revealed 10 phyla, 34 classes, 92 orders, 261 families, and 661 fungal genera. Across six of the seven substrates, Ascomycota was the most dominant phylum, with 60–90% of the relative abundance, followed by Basidiomycota with 5–37% of the relative abundance. However, an exception was observed in the cave wall surface (CW), where Basidiomycota was the dominant phylum, accounting for 67% of the relative abundance, followed by Ascomycota at 28% (Figure S2).

**Figure 4:**
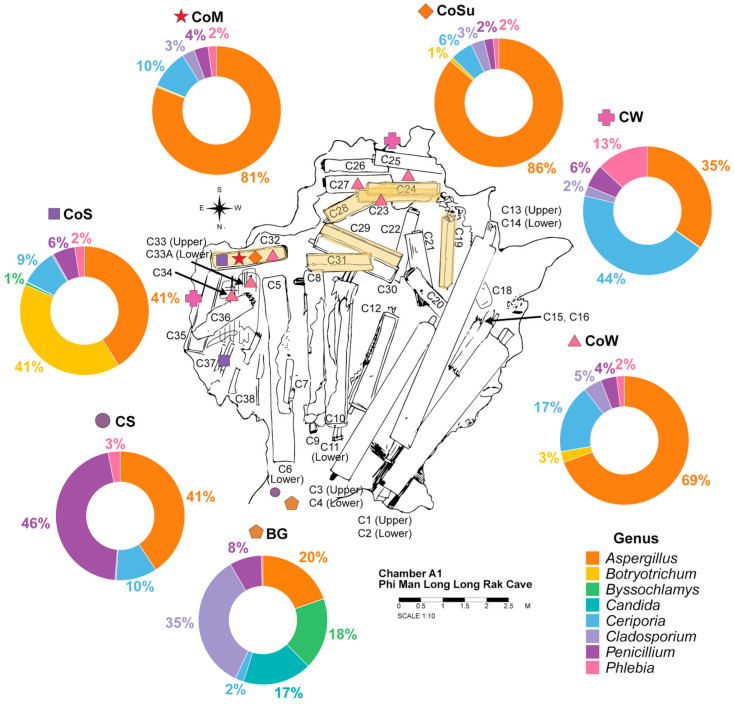
Pie chart showing the fungal community composition and relative abundance at the genus level across sample groups in Chamber A1. Each colored shape indicates the corresponding sample groups and their associated sampling locations, and the coffin highlighted in yellow shows fungal colonization. CS: cave soil, CoS: coffin soil, CW: cave wall, CoW: coffin wall, CoM: coffin mold, CoSu: artificial support, and BG: bat guano.

At the genus level, fungal community composition differed among substrates (Figure 4). In the cave soil (CS), *Penicillium* (46%) and *Aspergillus* (41%) were the most dominant genera. Similarly, in the coffin soil (CoS), *Aspergillus* (41%) remained dominant, along with *Botryotrichum* (41%). In the cave wall surface (CW), *Ceriporia* (44%) was the dominant genus, followed by *Aspergillus* (35%).

In contrast, coffin-associated substrates, including coffin wall surfaces (CoW), coffin wall surfaces with visible mold (CoM), and artificial support surfaces (CoSu), were overwhelmingly dominated by *Aspergillus*, accounting for 69%, 81%, and 86% of the relative abundance, respectively. In comparison, *Ceriporia* (2–17%) and *Penicillium* (2–4%) occurred only in minor proportions. The bat guano (BG) revealed a different and more diverse fungal genera compared to the other six substrates, with *Cladosporium* (35%) as the most abundant genus, followed by *Aspergillus* (20%), *Byssochlamys* (18%), *Candida* (17%), *Penicillium* (8%), and *Ceriporia* (2%). Moreover, PERMANOVA analysis further assessed similarities and differences in fungal community composition among substrate groups and revealed significant differences among groups (*p* = 0.0001).

Across the substrates, *Aspergillus* emerged as the most consistently dominant genus across multiple environmental substrates within the cave, particularly in coffin-associated areas, where it reached up to 86% relative abundance. It was also detected in surrounding soil, structural substrates, and artificial supports.

#### 3.3.3. Venn Diagram Analysis

**Figure 5:**
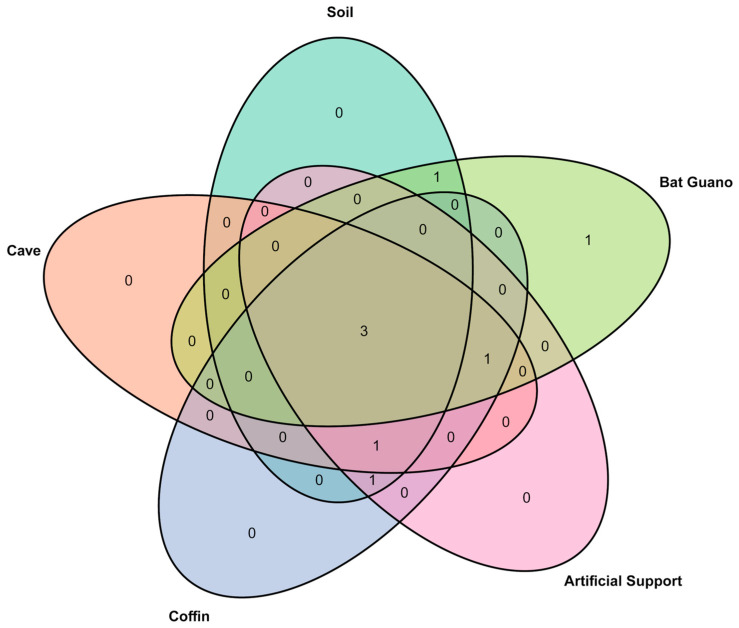
Venn diagram showing shared and unique fungal taxa among environmental substrates in Chamber A1. Numbers within overlapping regions represent the genus shared among five substrates: soil, cave wall surfaces, coffin wall surfaces, artificial support surfaces, and bat guano. Numbers in non-overlapping areas indicate substrate-specific genus. Three genera (*Aspergillus*, *Penicillium*, and *Ceriporia*) were shared across all five substrates. *Candida* was detected exclusively in bat guano. *Byssochlamys* was found in both soil and bat guano. *Botryotrichum* was shared among soil, coffin wall surfaces, and artificial support surfaces. *Phlebia* was shared among soil, cave wall surfaces, coffin wall surfaces, and artificial support surfaces. *Cladosporium* was shared among cave wall surfaces, coffin wall surfaces, artificial support surfaces, and bat guano.

Venn diagram analysis of five consolidated environmental groups revealed that *Aspergillus*, *Penicillium*, and *Ceriporia* were commonly found and shared across all groups in the chamber environment. In contrast, genera such as *Botryotrichum*, *Byssochlamys*, and *Candida* were specific to some groups. Notably, the soil group showed substantial overlap with multiple environmental location groups, particularly coffin-associated substrates such as coffins and artificial supports (Figure 5).

#### 3.3.4. Functional Prediction of Ecological Roles and Wood-Degrading Enzyme

**Figure 6:**
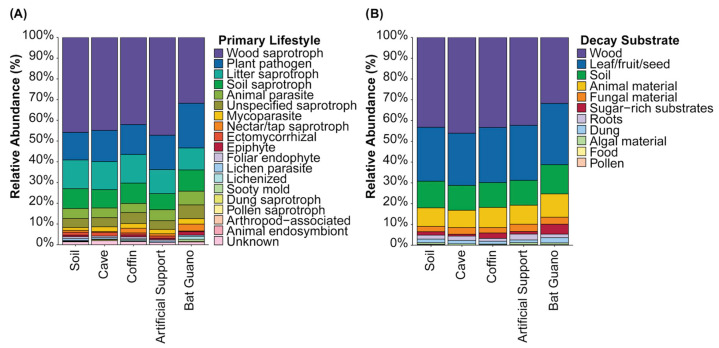
FungalTraits analysis of fungal communities in Chamber A1. (**A**) Primary lifestyle and (**B**) decay substrate.

The predictions from two functional tools provided complementary insights into the ecological and enzymatic capabilities of fungal communities across five consolidated environmental groups. Based on the FungalTraits analysis, two of the thirteen traits were assessed: primary lifestyle and decay substrates. For primary lifestyle, wood saprotrophs were the most abundant, accounting for 32–47% across all groups, with the highest proportion observed in the soil group (47%), followed by the cave (45%) and coffin groups (42%) (Figure 6A). In addition to wood saprotrophs, plant pathogens (13–22%), litter saprotrophs (11–14%), and soil saprotrophs (8–10%) were also present in notable proportions in all groups (Figure 6A). For decay substrates, fungi associated with wood were again the most abundant, representing 31–46% across all groups, with the highest value in the cave group (46%), closely followed by the soil and coffin groups (43%) (Figure 6B). For decay substrates, fungi associated with plant materials such as leaves, fruit, or seeds (25–29%), soil (12–14%), and animal materials (8–11%) were also detected (Figure 6B). Overall, wood-associated saprotrophs and wood-decay-related functional traits consistently dominated across environmental groups, particularly in the soil and coffin-associated groups.

Moreover, the PICRUSt2 analysis predicted 20 enzymes involved in wood modification and degradation, including cellulose-, hemicellulose-, lignin-, and lipid-degrading enzymes. Among all predicted enzymes, β-glucosidase was the most abundant across all environmental groups, followed by α-glucosidase and several hemicellulose-degrading enzymes, highlighting the predominance of polysaccharide (cellulose and hemicellulose) degradation potential within the fungal communities (Figure S3).

### 3.4. Commercial Fungicide Sensitivity Test

The commercial fungicide sensitivity test showed that after 24 h of incubation, all four concentrations (100%, 50%, 25%, and 12.5%) of the three selected commercial fungicides inhibited the fungal isolates, with larger inhibition zones generally observed at higher concentrations.

**Figure 7:**
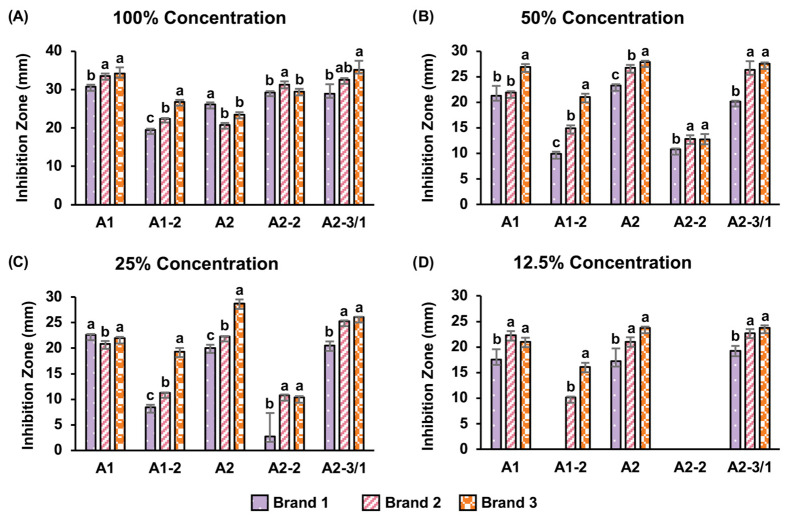
Inhibition zones (mm) of selected commercial anti-mold products on five fungal isolates at different concentrations: (**A**) 100%, (**B**) 50%, (**C**) 25%, and (**D**) 12.5%. A1: *Aspergillus terreus*, A1-2: *Aspergillus fischeri*, A2: *Aspergillus wentii*, A2-2: *Aspergillus terreus*, and A2-3/1: *Aspergillus sclerotiorum*. Different letters indicate statistically significant differences. Statistical significance was determined using a one-way ANOVA followed by Tukey’s HSD test, *p* < 0.05.

At 100% concentration, all fungicides produced a large clear inhibition zone against all five fungal isolates, indicating strong antifungal activity. Among them, Brand 3, which contains a mixture of fungicides as a component, including benzalkonium chloride, quaternary ammonium compounds, and 2-octyl-3-isothiazolinone (Table S3), exhibited the largest inhibition zones in most isolates, except for isolates A2 and A2-2 (Figure 7A and Figure S4, Table S6). In contrast, Brand 1 and Brand 2, which contain benzalkonium chloride as the sole fungicide (Table S3), showed slightly lower effectiveness (Figure 7A and Figure S4, and Table S6).

At 50% and 25% concentrations, all fungicides continued to inhibit all five fungal isolates; however, the inhibition zones were smaller compared to those observed at full strength. Brand 3 remained the most effective, followed by Brand 2 and Brand 1 (Figure 7B,C and Figure S4, and Table S6).

At a concentration of 12.5%, the inhibition zones became inconsistent. Some fungal isolates showed no inhibition, notably in isolate A2-2, where all fungicides were completely ineffective, and in isolate A1-2, where Brand 1 failed to inhibit the fungal isolates (Figure 7D and Figure S4, and Table S6).

## 4. Discussion

Caves are terrestrial environments with unique characteristics, such as being underground and oligotrophic (low in nutrients), which distinguish them from other above-ground terrestrial environments [19]. Beyond their ecological distinctiveness, caves possess substantial cultural and archaeological significance, with numerous artifacts discovered within them. Among these are wooden coffins commonly found in prehistoric burial caves. However, wooden coffins preserved in cave environments are vulnerable to deterioration caused by both abiotic and biotic factors [3,4,6,7]. Among the biotic factors, fungi play a critical role in weakening and degrading the wooden structures [3,4,6,7]. This study provides insight into the fungal community associated with wooden coffins and surrounding cave substrates, evaluates their biodeterioration potential, and examines potential environmental sources within a 2120-year-old prehistoric burial site, Phi Man Long Long Rak Cave, in northern Thailand. Understanding these processes is essential for developing strategies to mitigate biodeterioration and support the conservation and preservation of wooden coffins, the surrounding cave environment, and other wooden cultural heritage.

### 4.1. Effects of Environmental Parameters on Fungal Growth in Chamber A1

Temperature, humidity, and moisture are abiotic factors that affect fungal growth, survival, and colonization [17,38,39]. In caves, where conditions are typically dark, cool, and humid, temperature and humidity act as primary environmental filters that determine fungal viability and fungal growth [18,19,38,39]. These conditions also influence the materials preserved and stored within caves [18,39].

In Chamber A1 of Phi Man Long Long Rak Cave, environmental conditions recorded during both sampling periods in April 2023 and 2024 revealed that the temperature was approximately 23 °C and the relative humidity was around 70%. These conditions fall within the optimal range for fungal growth [9,39], particularly for wood-decaying fungi, thereby promoting colonization and wood biodeterioration [9,39,40]. In contrast, bacterial growth is generally favored under higher moisture conditions of 80% to 90% relative humidity and is more prominent in waterlogged or oxygen-limited environments such as buried or saturated wood [3,4]. Under the cave conditions observed here, fungi are therefore likely the primary drivers of biodeterioration, while bacteria co-occur and play a supportive rather than dominant role [3,4,5]. Accordingly, this study focuses on fungal-mediated wood biodeterioration. However, these observations are based on a single sampling period, and seasonal variation, particularly during the rainy season, may increase humidity and shift microbial dynamics, potentially enhancing the contribution of bacteria.

In addition to supporting fungal growth, humid conditions can also affect wooden substrates. As a porous material, wood can absorb atmospheric moisture in both liquid and vapor forms, increasing moisture content [18], which creates favorable conditions for fungal colonization and hyphal penetration, particularly for wood-decaying fungi [3,6,18,38,39,41].

Humidity and moisture appear to be particularly important in the southern part of the chamber, where rainwater dripping from the ceiling was observed [1,2]. Most coffins with visible fungal colonization were located in this area, and fungal growth was also detected on adjacent substrates, including surrounding soil and artificial support materials (Figure 2) [1,2]. This observation suggests that dripping water may increase localized humidity and temperature, thereby promoting fungal colonization [18,38,39]. Similar relationships between moisture availability and fungal abundance have been reported in soils [42], ancient tomb surfaces [43], cave surfaces [44], and wooden artifacts [6], indicating that wetter, humid environments may support higher fungal abundance, and potentially, greater colonization [18,38,39]. Collectively, the relatively stable temperature and high humidity in Chamber A1 likely provide permissive environmental conditions that facilitate fungal growth and colonization on both the cave and the wooden substrate.

### 4.2. Wooden Coffin Fungal Isolation and Potential Role in Wood Biodeterioration

Within the environmentally permissive conditions of the cave, fungal isolates obtained from the wooden coffins in this study were predominantly *Aspergillus*, including *A. terreus*, *A*. *fischeri*, *A*. *sclerotiorum*, and *A*. *wentii*, species previously reported and isolated from wooden artifacts and wood-related environments [6,45,46,47,48]. Species of *Aspergillus* are well known for producing diverse organic acids and extracellular enzymes, including wood-degrading enzymes, which contribute to the deterioration of wooden artefacts and heritage [3,6,8,21,49,50].

The biodeterioration potential of these fungi is particularly relevant considering the composition of the coffins. The coffins from Phi Man Long Long Rak Cave were constructed from teak (*Tectona grandis*), a durable hardwood composed primarily of cellulose, hemicellulose, lignin, and extractives (e.g., fats and waxes) [6,25]. Although teak is considered durable, prolonged exposure to the conditions in Chamber A1 may increase its susceptibility to fungal attack and colonization.

Wood biodeterioration occurs when fungi secrete extracellular enzymes that degrade lignocellulosic components [6,21]. In this study, biodeterioration assays revealed that four fungal isolates exhibited cellulolytic and hemicellulolytic activities, indicated by the production of cellulase and mannanase. These enzymes degrade cellulose and hemicellulose, respectively, and have previously been reported in the identified species [6,45,46,51,52,53,54,55,56,57]. This finding suggests that the cultured fungal isolates are associated with the degradation of cellulose and hemicellulose, components of the primary and secondary cell walls of wood [6], processes typically linked to early-stage wood biodeterioration [4,39,58]. Hemicellulose is often the first major wood component attacked and decomposed by fungi, and its degradation can significantly weaken the lignocellulosic structure of wood [4,6,39,58].

In contrast, ligninolytic and lipolytic activities were not detected in this study. However, this absence does not necessarily indicate that these fungi lack lignin-degrading potential. Several of the identified species, including *A*. *terreus*, *A*. *fischeri*, and *A*. *sclerotiorum*, have been reported to produce other ligninolytic enzymes, including lignin peroxidase and manganese peroxidase [6,45,51,52,53,57]. Therefore, examining a broader range of ligninolytic enzymes, together with additional cellulolytic and hemicellulolytic enzymes, would provide a more comprehensive evaluation of their potential role in lignocellulosic wood degradation [6,52,53].

Another important consideration is the limitation of culture-dependent methods. These methods often favor and are suitable for fast-growing and high-spore-forming fungi such as *Aspergillus* [21], whereas many fungi inhabiting specialized environments, including caves, may remain unculturable under laboratory conditions [59]. Consequently, the absence of ligninolytic and lipolytic activities in cultured isolates does not necessarily indicate that fungi capable of these functions are absent from the coffins or the cave environment.

In addition to enzymatic degradation, two fungal isolates produced acid, reducing the medium pH to approximately 3–4. Fungi can also produce various organic acids, including oxalic, malic, citric, succinic, fumaric, gluconic, acetic, propionic, and tartaric acids, which lower pH and create acidic conditions in wooden artifacts and substrates [49,50,60,61]. These acidic conditions can initiate early-stage wood biodeterioration by promoting the acid-catalyzed hydrolysis of cellulose and hemicellulose, dissolving and softening cell wall components, promoting the formation of cavities and surface cracks, and facilitating hyphal penetration and enzymatic activity, ultimately contributing to progressive structural damage [50,60,61].

Taken together, these findings demonstrate that fungal isolates from the wooden coffins possess the potential to contribute to wood deterioration through both enzymatic degradation and acid production, processes that can initiate and accelerate early-stage wood biodeterioration [4,6,39,50,58]. However, cultured isolates represent only a subset of the total fungal community associated with the coffins, and the limitations of culture-dependent methods underscore the importance of examining the broader fungal community structure. Therefore, further investigation of the overall fungal communities within the cave and coffin-associated environments is essential to better understand the fungal communities potentially involved in wooden coffin biodeterioration.

### 4.3. Cave Fungal Communities, Functional Potential, and Environmental Source Associated with Wooden Coffin Biodeterioration

Culture-independent analysis revealed that fungal communities in Chamber A1 were dominated by *Aspergillus*, *Penicillium*, *Cladosporium*, and *Ceriporia*, the most abundant genera across the environmental substrate groups. Notably, *Aspergillus*, *Penicillium*, and *Ceriporia* were shared across all environmental groups, suggesting the presence of a core fungal taxa within the cave chamber environments.

The dominance of *Aspergillus* and *Penicillium* in this cave environment is not unexpected, as these genera are well known for their adaptability, including high spore production and tolerance to fluctuating temperature and moisture conditions [6,10,16,21,62]. These genera are frequently reported as dominant in fungal communities on cave substrates such as soil, rock surfaces, and cave wall surfaces worldwide, including in Southern Thailand [9], China [11,12,13,14,15], India [63], Malaysia [64], Italy [62], Serbia [65], and Slovakia [66]. In addition to cave environments, both genera are widely detected on wooden artifacts and heritage, highlighting their potential role in biodeterioration processes [3,6,10,22,67]. Similarly, *Ceriporia* has been reported in cave rock surfaces in Southern Thailand [9] and in cave soils in China [11], consistent with its predominance on cave walls and its detection in soil and wooden substrates in this study. However, differences in community composition can be observed among cave environments, which are likely influenced by site-specific factors, including geographic location, environmental conditions, substrate type, organic matter availability, and levels of human disturbance, such as whether caves are open or closed to visitors [38,39]. Beyond the shared genera, this study also reveals site-specific fungal genera. *Phlebia* was detected in this study, but is rarely reported from the previously mentioned cave environments. Its presence is likely due to the presence of wooden coffins and fragmented wood in this study, which provide suitable niches for wood-decaying fungi [68,69]. Although *Ceriporia* has been reported in some cave environments, such as those in Southern Thailand [9] and China [11], its occurrence is inconsistent across other caves. The presence of *Ceriporia* in these sites may be associated with similar environmental conditions or substrates, such as wood-derived organic matter, which could influence its distribution.

Among the detected genera, *Aspergillus* was particularly dominant in soil and coffin-associated substrates, while *Penicillium* was also detected, especially in cave soil (CS). Moreover, culture-dependent isolation further confirmed the presence of *Aspergillus* species in wooden coffin samples and demonstrated their enzymatic and acid-producing capabilities, supporting their potential role in coffin colonization and biodeterioration. The dominance of *Aspergillus* in culture-dependent analyses and its high abundance in culture-independent analyses indicate a complementary agreement between these two methods. *Aspergillus* was consistently detected by both approaches. Meanwhile, culture-independent analysis identified additional genera, including *Penicillium* and *Ceriporia*, within the fungal community of wooden coffins that were not fully recovered through culture-dependent isolation. This outcome reflects methodological differences in fungal detectability. Culture-dependent methods only isolate fungi that can be cultivated in vitro, whereas culture-independent methods provide a more comprehensive assessment of the overall fungal community.

Both *Aspergillus* and *Penicillium* species are commonly known as soil-inhabiting fungi that are frequently found on wooden artifacts and cultural heritage [3,6,67]. These fungi are classified as soft-rot fungi capable of degrading cellulose and hemicellulose, components of the primary and secondary cell walls of wood, through enzymatic and acid-producing capabilities [3,6,17,21,60,67]. Soft-rot decay is generally more common in hardwoods than in softwoods, which is particularly relevant in this study because the wooden coffins were constructed from teak, a hardwood species [67]. In particular, *Aspergillus* species are known to cause both Type I and Type II soft-rot decay, characterized by cavity formation and the erosion of wood cell walls [3,6,67].

In contrast, *Ceriporia*, detected on several substrates and particularly abundant on cave walls, is a white-rot fungus capable of degrading lignin in addition to cellulose and hemicellulose [70]. Its presence, therefore, suggests the potential of fungi capable of more advanced lignin breakdown and wood degradation within the cave environment [6,7,39].

Beyond *Aspergillus* dominance in soil, *Botryotrichum* was also detected specifically in coffin soil. This genus is a soil fungus commonly associated with organic-rich soils, agricultural land, and animal dung [71,72], suggesting that the soil surrounding the coffins may be enriched with organic matter, possibly derived from decaying wood fragments, wood litter, or nutrient inputs from cave animals and arthropods. These findings highlight the dynamic and nutrient-active nature of the soil environment adjacent to the coffins.

Importantly, most wooden coffins are placed directly on the cave floor or close to the soil, while only some are supported by artificial structures. Even where supports are present, fungal colonization was observed on surrounding soil, artificial supports, and adjacent wooden surfaces [1,2]. These observations indicate that fungi are not restricted to coffin substrates but may disperse among nearby materials within the cave environment through direct contact or short-distance transfer, including colonization of adjacent substrates [3,7,10,47,67]. The presence of shared fungal genera between soil and coffin-associated substrates indicates ecological connectivity at the soil–wood interface, particularly where direct contact occurs.

Moreover, the fungal isolates obtained from wooden coffins in this study, including *Aspergillus terreus*, *A*. *fischeri*, *A*. *wentii*, and *A*. *sclerotiorum*, are not only associated with wood but are also well-known soil-inhabiting fungi commonly associated with soil, decaying plant material, and other organic substrates [47,48,57,73,74,75]. Their occurrence in both soil and coffin samples, therefore, supports the possibility that coffin-associated fungi originate from surrounding soil–coffin environments.

Although many genera were shared among the soil and coffin-associated substrate groups, another cave substrate examined in this study, bat guano (BG), exhibited a more distinct fungal community, characterized by the dominance of *Cladosporium* and the unique detection of *Candida*. This pattern likely reflects the ecological role of bats, as the fungi detected in guano may originate from their diet and food sources, which mostly come from external environments [64,76]. Therefore, bats can introduce fungal spores into caves through their diet and dung, as well as their bodies or cadavers. In addition, guano deposits and bat carcasses provide nutrient-rich organic matter that can enhance fungal growth and increase fungal diversity within the cave [64,77,78,79].

*Cladosporium* and *Candida* are commonly associated with bat habitats and frequently detected in guano deposits due to their ability to exploit nutrient-rich organic substrates [80,81]. *Cladosporium* is widely detected in soil and air, and its conidia readily disperse through air currents or disturbances caused by bat activity [13,80], with some species also producing cellulolytic and hemicellulolytic enzymes involved in wood polysaccharide degradation [82]. Yeasts such as *Candida* are also frequently detected in nutrient-rich substrates, including bat guano, and may contribute to the initial breakdown of organic substrates and nutrient mineralization [81]. Consequently, bat-guano-associated fungi may facilitate fungal dispersal and influence colonization across cave substrates, including wooden coffins, potentially contributing to wood biodeterioration.

However, when interpreting the overall fungal community structure in Chamber A1, it is essential to recognize that it may include both ancient or native cave fungi and taxa introduced from external environments. Fungal spores can enter caves through multiple pathways, including airborne dispersal via wind [13,83]. Additionally, percolating water, such as rainwater dripping and ceiling drips, may transport spores from external environments into the cave interior, particularly in areas directly exposed to water flow [38,41,83,84]. Beyond abiotic vectors such as air and water, biological vectors, including cave-dwelling arthropods and animals such as bats, may further facilitate fungal dispersal into caves [64,76,77,79]. Moreover, human activities, particularly during cave discovery and archaeological exploration, may also introduce external fungi through equipment, clothing, or footwear. Therefore, comparative analyses between cave and external environments are necessary to distinguish resident taxa from introduced species and to better understand the origin and dynamics of fungal community assembly in cave environments.

Functional trait and enzyme predictions further supported the potential role of fungal communities in wood deterioration. Functional trait analysis revealed that fungal communities within Chamber A1 were dominated by a wood-saprotrophic lifestyle involved in the decomposition of wood and plant materials, while coffin samples also exhibited a high level of this. This pattern likely reflects the ecological adaptation of cave fungi to utilize lignocellulosic substrates, where materials like intact wooden coffins and broken wood debris act as the main organic nutrient sources in the cave environment [85,86,87]. These traits also directly contribute to the biodeterioration of wooden coffins.

Consistent with this pattern, enzyme predictions also identified multiple wood-degrading enzymes, particularly those involved in cellulose and hemicellulose degradation, supporting the culture-dependent results that demonstrate the capacity of isolated fungi to degrade wood polysaccharides (cellulose and hemicellulose) associated with the early stages of wood biodeterioration [4,8,10,39,58]. In addition, predicted ligninolytic and lipolytic enzymes were also detected, suggesting the potential degradation of lignin and wood extractives. This is particularly noteworthy because ligninolytic and lipolytic activities were not detected in culture-dependent results, highlighting that culture-dependent methods represent only a subset of the functional potential present in situ, whereas culture-independent methods reveal a broader functional metabolic capability.

Nevertheless, functional predictions based on FungalTraits and PICRUSt2 should be interpreted with caution, as they rely on genus-level assignments from ITS data, which limit species-level accuracy and may overlook functional variation within genera [88]. This can mask rare but functionally important taxa, while unclassified or uncultured groups remain unassigned due to database limitations [88,89]. In addition, PICRUSt2 was originally developed for 16S rRNA data, and its application to fungal ITS data is constrained by the limited number of reference genomes [89,90]. Therefore, these predictions should be considered indicative rather than definitive, and ideally validated using targeted enzyme assays or shotgun metagenomics for more robust functional insights [89,90].

Collectively, the predominance of *Aspergillus*, together with other wood-degrading genera such as *Penicillium* and *Ceriporia*, and the presence of wood-saprotrophic traits across both soil and coffin-associated substrates, compellingly indicate shared fungal communities at the soil–coffin interface. Fungi are distributed throughout the cave, particularly in coffin-associated materials and in adjacent substrates in direct contact with soil, further supporting this association. This conclusion is corroborated by the isolation of *Aspergillus* species, which are recognized as soil- and wood-inhabiting fungi and demonstrated cellulolytic and hemicellulolytic activities in this study. However, these patterns do not permit the determination of the direction of fungal dispersal or the identification of a primary source based on the current data. It is equally plausible that soil-associated fungi contribute to the colonization of wooden substrates, that fungi originating from colonized wood accumulate in the surrounding soil, or that bi-directional exchange occurs between these substrates. Therefore, these findings are best interpreted as evidence of interconnected fungal communities at the soil–wood interface, rather than as an indication of a single origin or reservoir of wood-colonizing fungi.

In the context of fungal decay succession, the dominance of *Aspergillus*, a soft-rot fungus, together with other core fungal genera such as *Penicillium*, another soft-rot fungus, and *Ceriporia,* a white-rot fungus, suggests that the wooden coffins in Chamber A1 are undergoing early to intermediate stages of deterioration. Early-stage deterioration is typically characterized by the surface colonization and initial degradation of cellulose and hemicellulose [6,39,58], whereas late-stage deterioration involves extensive lignin degradation and structural collapse [3,6,7,39]. These findings emphasize the urgency of fungal control strategies. If left unmanaged, continued fungal activity under stable cave environmental conditions may accelerate deterioration toward irreversible advanced deterioration. Therefore, effective strategies to control or eliminate fungi in the cave environment, particularly in soil, which likely serves as a potential source of wood-degrading fungi, and on coffin surfaces, are urgently needed to preserve these archaeological wooden coffins.

### 4.4. Fungal Control Strategies for Wooden Coffin Preservation in Cave Environments

In recent years, the use of chemical preservation methods has become an alternative method for protecting cultural heritage materials and archaeological artifacts. In this study, fungicides commonly used as active ingredients in commercial surface-cleaning products, including benzalkonium chloride, 2-octyl-3-isothiazolinone, and quaternary ammonium compounds mixtures, were evaluated for their antifungal activity [91,92].

All tested fungicides inhibited fungal growth in vitro, with products containing mixtures of fungicide active components showing greater inhibition, even at sub-maximal concentrations. This suggests potential additive or synergistic effects and supports conservation and ecological considerations, as minimizing fungicide or chemical use is critical to reduce risks to cave environments, including substrate damage, chemical accumulation, and disruption of non-target microbial communities [8,92].

Benzalkonium chloride and quaternary ammonium compounds function as cationic surfactants with a positive charge, enabling strong interactions with the negatively charged surfaces of microbial cells. These interactions disrupt the fungal phospholipid bilayer, increasing membrane permeability and promoting emulsification and denaturation of structural proteins [93,94]. In contrast, 2-octyl-3-isothiazolinone acts as a fungicide, disrupting fungal cell walls by diffusing across them and reacting with thiol-containing groups in cellular components, disrupting enzymatic functions and leading to cellular growth inhibition and death [95]. Therefore, the combination of cell wall and cell membrane disruption and intracellular enzyme inactivation likely explains the superior inhibition observed for the mixed fungicide components.

However, the assays conducted in this study were performed under in vitro laboratory conditions, which cannot fully replicate the complexity and heterogeneity of cave ecosystems that involve diverse microbial communities, microbial interactions, biofilm formation, environmental fluctuations, nutrient dynamics, and continuous propagule input from surrounding substrates [21,37,91,96]. In particular, if the soil–coffin interface acts as the potential reservoir for coffin-colonizing fungi, treating coffin surfaces alone may not prevent recurrent colonization, and applying these inhibition results must be approached with caution.

Therefore, the fungicide assays conducted in this study should be considered preliminary insights into potential fungal control strategies. Further studies are required to evaluate the effectiveness, ecological impacts, and long-term sustainability of chemical fungicide applications before any practical implementation in situ or in cave environments. Consequently, the development of practical and sustainable preservation strategies for wooden coffins in cave environments will require further investigation.

## 5. Conclusions

This study investigated fungal communities associated with the biodeterioration of ancient wooden coffins at a 2120-year-old prehistoric site, Phi Man Long Long Rak Cave, northern Thailand, integrating environmental measurements, culture-dependent and culture-independent approaches, functional predictions, and fungicide sensitivity testing. The stable temperature and sustained humidity create favorable conditions for fungal colonization and metabolic activity, facilitating continuous wood deterioration. Culture-dependent analyses revealed *Aspergillus* isolates from wooden coffin samples, with cellulolytic, hemicellulolytic, and acid-producing capacities, indicating their involvement in the degradation of structural polysaccharides and early-stage biodeterioration. However, these isolates represent only the culturable fraction of the fungal community. Culture-independent analyses identified *Aspergillus* as the dominant genus, especially in soil and coffin-associated substrates, with *Penicillium*, *Cladosporium*, and *Ceriporia* also frequently detected. Functional predictions indicated a predominance of wood-saprotrophic fungi. The compositional similarity between soil and coffin-associated substrates, together with shared wood-degrading taxa and wood-saprotrophic abilities, supports ecological connectivity across soil–wood interfaces in the cave environment. However, the current data do not permit the determination of fungal dispersal directionality or the identification of a primary source. Both soil-to-wood and wood-to-soil dispersal pathways remain plausible, indicating the bidirectional exchange and ecological connectivity of lignocellulolytic fungi between these substrates. Direct source-tracking is required to confirm these dispersal pathways. All commercial fungicides inhibited the isolates, with the mixed fungicides showing the highest efficacy; however, laboratory inhibition does not necessarily reflect in situ performance. However, this study was based on limited sampling during two April sampling campaigns and may not fully capture seasonal or interannual variation in cave microclimate and fungal community dynamics. Overall, these findings enhance understanding of fungal biodeterioration processes in prehistoric wooden coffins and provide a foundation for future functional validation and conservation strategies in cave heritage environments.

## Figures and Tables

**Table 1:** GenBank accession numbers and BLAST results revealed the highest sequence similarity between the fungal isolates in this study and the holotype species available in the GenBank database.

*Aspergillus* Isolates	Loci	GenBank Accession Number	Closely Related Holotype Fungal Species/Similarity Value (%)
A1	ITS	PZ356817	*Aspergillus terreus* NRRL 255/99.83
	*BenA*	PZ363894	*Aspergillus terreus* NRRL 255/100
	*CaM*	PZ363889	*Aspergillus terreus* NRRL 255/100
A1-2	ITS	PZ356818	*Aspergillus fischeri* NRRL 181/100
	*BenA*	PZ363895	*Aspergillus fischeri* NRRL 181/100
	*CaM*	PZ3638890	*Aspergillus fischeri* NRRL 181/100
A2	ITS	PZ356849	*Aspergillus wentii* CBS 104.07/100
	*BenA*	PZ363896	*Aspergillus wentii* CBS 104.07/100
	*CaM*	PZ3638891	*Aspergillus wentii* CBS 104.07/100
A2-2	ITS	PZ356850	*Aspergillus terreus* NRRL 255/99.83
	*BenA*	PZ363897	*Aspergillus terreus* NRRL 255/100
	*CaM*	PZ3638892	*Aspergillus terreus* NRRL 255/100
A2-3/1	ITS	PZ356863	*Aspergillus sclerotiorum* NRRL 415/100
	*BenA*	PZ363898	*Aspergillus sclerotiorum* NRRL 415/100
	*CaM*	PZ3638893	*Aspergillus sclerotiorum* NRRL 415/100

**Table 2:** Summary of enzyme activity of fungal isolates.

*Aspergillus* Isolates	Wood Degrading Enzymes			
	Cellulolytic (Cellulase)	Hemicellulolytic (Mannanase)	Ligninolytic (Laccase)	Lipolytic (Lipase)
*A. terreus* A1	+	+	−	−
*A. fischeri* A1-2	+	+	−	−
*A. wentii* A2	+	+	−	−
*A. terreus* A2-2	+	+	−	−
*A. sclerotiorum* A2-3/1	−	−	−	−

Note: ‘+’ denotes positive enzyme activity; ‘−’ denotes negative enzyme activity.

**Table 3:** Summary of acid production of fungal isolates.

*Aspergillus* Isolates	Acid Production
*A. terreus* A1	+
*A. fischeri* A1-2	−
*A. wentii* A2	−
*A. terreus* A2-2	−
*A. sclerotiorum* A2-3/1	+

Note: ‘+’ denotes acid production (pH lower than control); ‘−’ denotes no acid production (pH higher than control).

## Data Availability

The data presented in this study are publicly available. The data can be accessed under the following accession numbers: PZ356817, PZ363894, and PZ363889 for fungal isolate A1; PZ356818, PZ363895, and PZ3638890 for fungal isolate A1-2; PZ356849, PZ363896, and PZ3638891 for fungal isolate A2; PZ356850, PZ363897, and PZ3638892 for fungal isolate A2-2; and PZ356863, PZ363898, and PZ3638893 for fungal isolate A2-3/1. The BioProject accession number PRJNA1310159 for the ITS amplicon sequencing data of seven environmental substrate groups.

## Supplementary Materials

The following supporting information can be downloaded at: https://www.mdpi.com/article/10.3390/jof12050380/s1, Figure S1: Rarefaction curves of fungal amplicon sequence variants (ASVs) across all samples from Chamber A1; Figure S2: Stacked bar chart showing fungal community composition and relative abundance at the phylum level across sample groups from Chamber A1; Figure S3: Heatmap of 20 enzymes involved in wood modification and degradation, predicted using PICRUSt2; Figure S4: Inhibition zones (mm) of selected commercial fungicide products on the growth of a fungal isolate on half-strength PDA at four different concentrations; Table S1: Detailed information on seven environmental substrate and wooden coffin samples in Chamber A1 of Phi Man Long Long Rak Cave; Table S2: Detailed composition of the basal medium; Table S3. Detailed of the commercial fungicide component; Table S4. Detailed pH measurements of growth medium inoculated with fungal isolates; Table S5. Detailed of fungal amplicon sequence variant (ASV) counts at each processing step from QIIME2 analysis of fungal associated with Chamber A1 of Phi Man Long Long Rak Cave; Table S6: Detailed inhibition zones (mm) measurements of selected commercial fungicide products on fungal isolates.
