# Supplementary material for: Fungal Communities Associated with Wooden Coffins in a Prehistoric Burial Cave
Source: J Fungi (Basel). 2026 May 21;12(5):380. doi: 10.3390/jof12050380 (PMC13208523; doi:10.3390/jof12050380)
Supplement: Supplementary file 1 [file jof-12-00380-s001.zip › Revised-Supplementary.pdf]

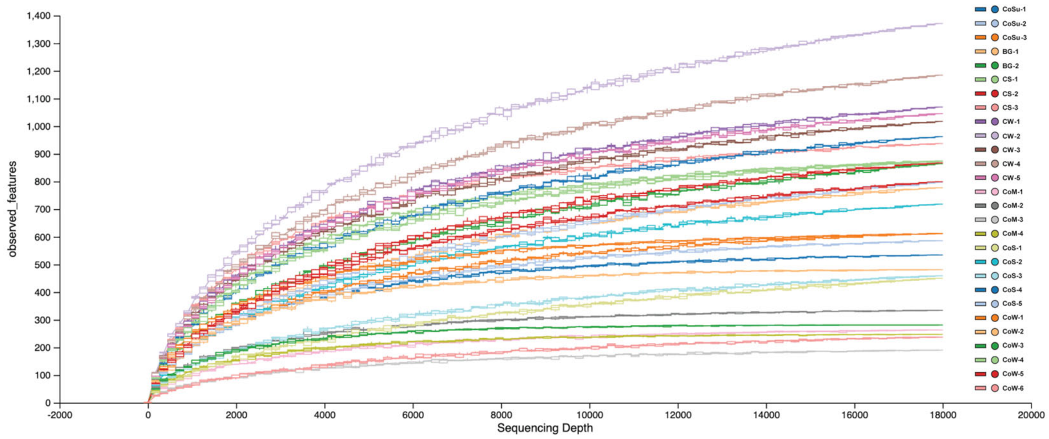

Figure S1. Rarefaction curves of fungal amplicon sequence variants (ASVs) across all samples from Chamber A1. Each curve represents a different sample, distinguished by color. CS: Cave soil, CoS: Coffin soil, CW: Cave wall, CoW: Coffin wall, CoM: Coffin mold, CoSu: Artificial support, and BG: Bat guano.

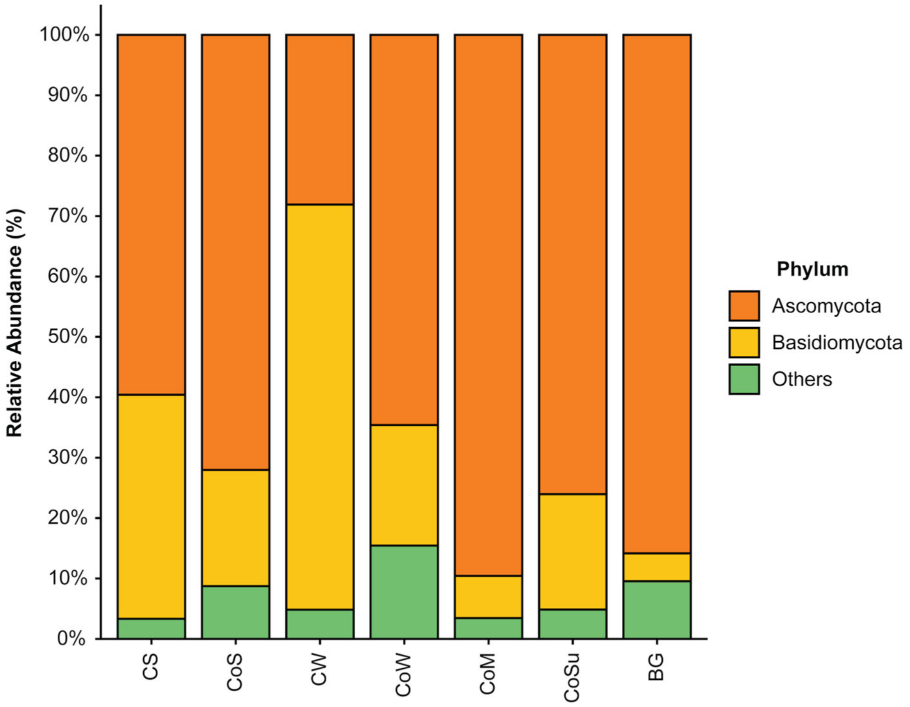

Figure S2. Stacked bar chart showing fungal community composition and relative abundance at the phylum level across sample groups from Chamber A1. CS: Cave soil, CoS: Coffin soil, CW: Cave wall, CoW: Coffin wall, CoM: Coffin mold, CoSu: Artificial support, and BG: Bat guano.

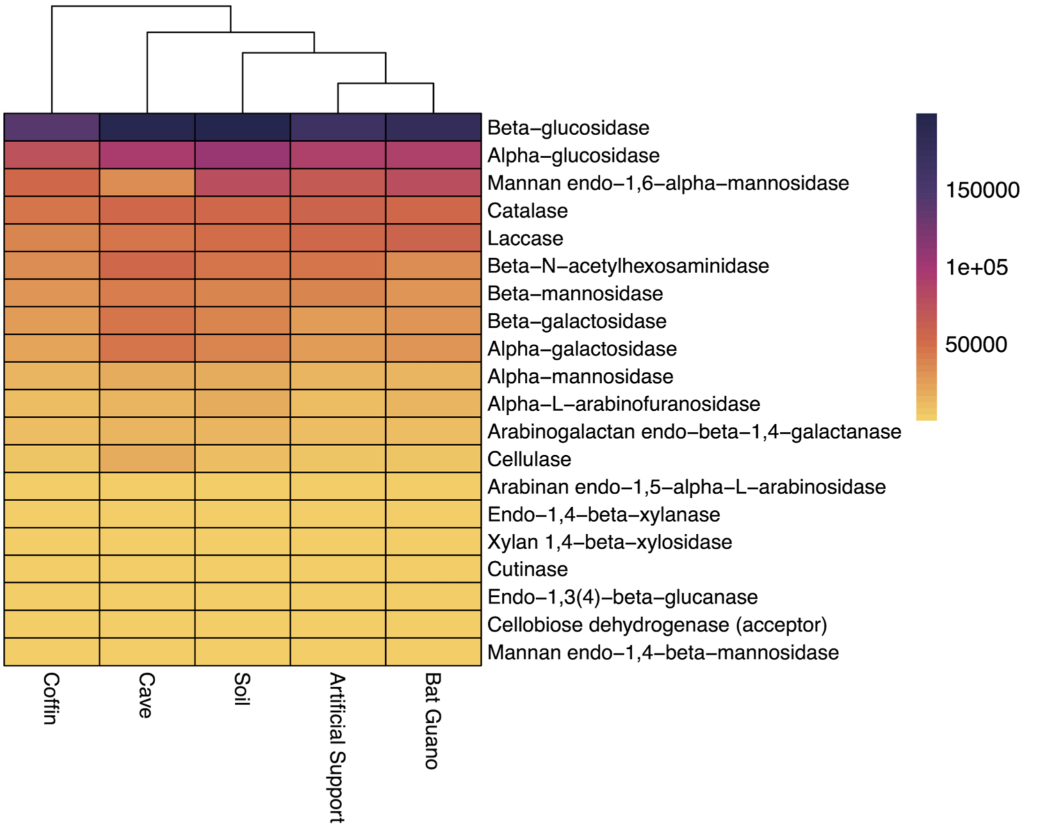

Figure S3. Heatmap of 20 enzymes involved in wood modification and degradation, predicted using PICRUSt2. The color gradient from dark purple to yellow represents enzyme abundance, with dark purple indicating the highest abundance and yellow indicating the lowest abundance, in each environment group from Chamber A1.

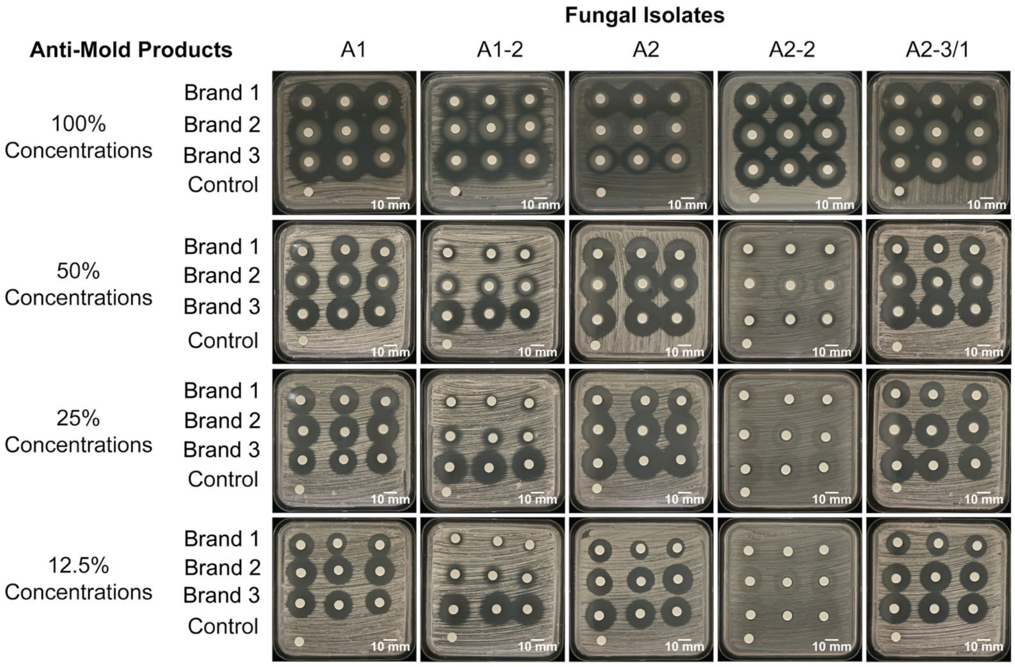

Figure S4. Inhibition zones (mm) of selected commercial fungicide products on the growth of a fungal isolate on half-strength PDA at four different concentrations. A1: *Aspergillus terreus*, A1-2: *Aspergillus fischeri*, A2: *Aspergillus wentii*, A2-2: *Aspergillus terreus*, and A2-3/1: *Aspergillus sclerotiorum*.

Table S1. Detailed information on seven environmental substrate and wooden coffin samples in Chamber A1 of Phi Man Long Long Rak Cave.

Wooden Coffin Samples	Coffin Number	Location	Sample Number
**Wooden Coffin Fragments**			
Coffin Lid	C32	The coffin fragment	-
Coffin Body	C32	The coffin fragment	-
Coffin Tag Paper	C32	The coffin tag paper	-
**Environmental Substrates**	**Coffin Number**	**Location**	**Sample Number**
**Cave Soil (CS)**			
1	Soil at the chamber entrance area	Entrance area	3
		**Total**	**3**
**Coffin Soil (CoS)**			
1	C23	The soil within the coffin	3
2	C37	The soil within the coffin	2
		**Total**	**5**
**Cave Wall (CW)**			
1	The eastern wall of the chamber wall near C35	Cave wall	3
2	The southern wall of the chamber near C25 and C26	Cave wall	2
		**Total**	**5**
**Coffin Wall (CoW)**			
1	C23	The interior surface	1
2	C24	The exterior surface	1
3	C27	The interior surface	1
4	C32	The interior surface	1
5	C33	The interior surface	1
6	C34	The exterior surface	1
		**Total**	**6**
**Coffin Mold (CoM)**			
1	C32	The exterior surface with mold	4
		**Total**	**4**
**Artificial Support (CoSu)**			
1	Nylon 6,6 support of C32	The exterior surface with mold	3
		**Total**	**3**
**Bat Guano (BG)**			
1	Bat guano at the chamber entrance area	Bat guano	2
		**Total**	**2**

Table S2. Detailed composition of the basal medium.

Component	Basal Medium
KH_2PO_4,	1.36 g/L
$MgSO_4.7H_2O$	0.20 g/L
NaCl	2.00 g/L
$(NH_4)_2SO_4$	1.00 g/L
$FeSO_4.7H_2O$	0.01 g/L
Yeast extract	0.10 g/L
Agar powder	20.00 g/L
**Cellulase**<br>Carboxymethyl cellulose (CMC)	5.00 g/L
**Mannanase**<br>Locust bean gum	5.00 g/L
**Laccase**<br>Guaiacol	0.20 mL/L
**Lipase**<br>Tween 20	10.00 mL/L

Table S3. Detailed of the commercial fungicide component.

Commercial Fungicides	Component
Brand 1	Benzalkonium chloride 1.4%
Brand 2	Benzalkonium chloride 1%–10%
Brand 3	Benzalkonium chloride,<br>Quaternary ammonium compounds,<br>and 2-octyl-3-isothiazolinone

Table S4. Detailed pH measurements of growth medium inoculated with fungal isolates.

***Aspergillus* Isolates**	**pH Value (Mean ± SD)**
Control	5.81 ± 0.07
Aspergillus terreus A1	3.40 ± 0.17
Aspergillus fischeri A1-2	6.04 ± 0.80
Aspergillus wentii A2	5.85 ± 0.05
Aspergillus terreus A2-2	6.21 ± 0.05
Aspergillus sclerotiorum A2-3/1	4.30 ± 0.32

Table S5. Detailed of fungal amplicon sequence variant (ASV) counts at each processing step from QIIME2 analysis of fungal associated with Chamber A1 of Phi Man Long Long Rak Cave.

**1. Demultiplexed Sequence Counts Summary**	
	Reads
Minimum	29,640
Maximum	159,417
Total	2,616,073
**2. After Denoising, Quality Filtering, and Low-Quality Reads and Chimeric Sequences Removal**	
Table Summary	
	Sample
Number of samples	28
Number of features	9,444
Total frequency	1,955,305
Frequency per Sample	
	Frequency
Minimum frequency	17,915.00
1st quartile	59,235.75
Median frequency	66,690.50
3rd quartile	89,097.25
Maximum frequency	120,170.00
Mean frequency	69,832.32
**3. After Singleton Removal**	
Table Summary	
	Sample
Number of samples	28
Number of features	9,440
Total frequency	1,955,301
Frequency per Sample	
	Frequency
Minimum frequency	17,915.00
1st quartile	59,235.75
Median frequency	66,690.50
3rd quartile	89,096.50

Maximum frequency	120,169.00
Mean frequency	69,832.18
**4. After Taxonomic Classification and Rarefaction Analysis**	
Table Summary	
	Sample
Number of samples	28
Number of features	7,892
Total frequency	501,620
Frequency per Sample	
	Frequency
Minimum frequency	17,915.00
1st quartile	17,915.00
Median frequency	17,915.00
3rd quartile	17,915.00
Maximum frequency	17,915.00
Mean frequency	17,915.00
Frequency per Feature	
	Frequency
Minimum frequency	1.00
1st quartile	2.00
Median frequency	3.00
3rd quartile	10.00
Maximum frequency	23,972.00

Table S6. Detailed inhibition zones (mm) measurements of selected commercial fungicide products on fungal isolates.

Aspergillus Isolates	Concentration	Anti-Mold Products	Inhibition Zone (mm) (Mean ± SD)
Aspergillus terreus A1	100%	Brand 1	30.70 ± 0.57
		Brand 2	33.53 ± 0.61
		Brand 3	34.16 ± 1.65

	50%	Brand 1	21.31 ± 1.91
		Brand 2	21.89 ± 0.28
		Brand 3	26.93 ± 0.57
	25%	Brand 1	22.60 ± 0.15
		Brand 2	20.87 ± 0.53
		Brand 3	21.94 ± 0.24
	12.5%	Brand 1	17.54 ± 2.01
		Brand 2	22.27 ± 0.85
		Brand 3	21.01 ± 0.82
Aspergillus fischeri A1-2	100%	Brand 1	19.44 ± 0.34
		Brand 2	22.42 ± 0.19
		Brand 3	26.83 ± 0.49
	50%	Brand 1	9.93 ± 0.32
		Brand 2	14.90 ± 0.62
		Brand 3	21.00 ± 0.71
	25%	Brand 1	8.41 ± 0.51
		Brand 2	11.29 ± 0.08
		Brand 3	19.28 ± 0.79
	12.5%	Brand 1	ND
		Brand 2	10.15 ± 0.20
		Brand 3	16.10 ± 0.81
Aspergillus wentii A2	100%	Brand 1	26.10 ± 0.61
		Brand 2	20.76 ± 0.53
		Brand 3	23.57 ± 0.64
	50%	Brand 1	23.27 ± 0.25
		Brand 2	26.76 ± 0.56
		Brand 3	27.89 ± 0.23
	25%	Brand 1	20.06 ± 0.61
		Brand 2	22.30 ± 0.09
		Brand 3	28.70 ± 0.85
	12.5%	Brand 1	17.23 ± 2.47

		Brand 2	21.03 ± 0.86
		Brand 3	23.73 ± 0.25
Aspergillus terreus A2-2	100%	Brand 1	29.30 ± 0.25
		Brand 2	31.30 ± 0.84
		Brand 3	29.49 ± 0.67
	50%	Brand 1	10.78 ± 0.16
		Brand 2	12.85 ± 0.71
		Brand 3	12.77 ± 1.02
	25%	Brand 1	8.05 ± 0.25
		Brand 2	10.74 ± 0.24
		Brand 3	10.36 ± 0.24
	12.5%	Brand 1	ND
		Brand 2	ND
		Brand 3	ND
Aspergillus sclerotiorum A2-3/1	100%	Brand 1	29.90 ± 2.51
		Brand 2	32.53 ± 0.52
		Brand 3	35.15 ± 2.39
	50%	Brand 1	20.17 ± 0.18
		Brand 2	26.36 ± 1.70
		Brand 3	27.55 ± 0.27
	25%	Brand 1	20.51 ± 0.79
		Brand 2	25.29 ± 0.15
		Brand 3	26.04 ± 0.12
	12.5%	Brand 1	19.23 ± 1.02
		Brand 2	22.74 ± 0.79
		Brand 3	23.74 ± 0.52

Note: 'ND' means not detected.
